# (6*Z*)-3,5-Bis(4-fluoro­phen­yl)-6-(1-hy­droxy­ethyl­idene)cyclo­hex-2-en-1-one

**DOI:** 10.1107/S1600536812003078

**Published:** 2012-02-10

**Authors:** Jerry P. Jasinski, James A. Golen, S. Samshuddin, B. Narayana, H. S. Yathirajan

**Affiliations:** a Department of Chemistry, Keene State College, 229 Main Street, Keene, NH 03435-2001, USA; b Department of Studies in Chemistry, Mangalore University, Mangalagangotri 574 199, India; c Department of Studies in Chemistry, University of Mysore, Manasagangotri, Mysore 570 006, India

## Abstract

In the title compound, C_20_H_16_F_2_O_2_, the cyclo­hex-2-en-1-one ring adopts a distorted envelope conformation and the dihedral angles between its six-atom mean plane and the fluorophenyl rings are 38.9(8) and 82.3(1)°. The two fluoro­phenyl rings are oriented at an angle of 77.3 (3)°. The long hy­droxy O—H bond length of 1.22 (3) and the H⋯O distance of 1.28 (3) Å, together with a longer than expected C=O bond length [1.290 (2) Å] in the hy­droxy(en-1-one) group, indicate sharing of the H atom as O⋯H⋯O between the two O atoms and the influence of electron delocalization. Weak C—H⋯O inter­molecular inter­actions form an infinite two-dimensional network in (011).

## Related literature

For biological applications of some cyclo­hexenones, see: Eddington *et al.* (2000 ▶); Kolesnick & Golde (1994 ▶). For background to the applications of cyclo­hexenones, see: Padmavathi *et al.* (1999 ▶, 2000 ▶); Padmavathi, Sharmila, Somashekara Reddy & Bhaskar Reddy (2001 ▶); Padmavathi, Sharmila, Balaiah *et al.* (2001 ▶). For related structures, see: Fischer *et al.* (2008 ▶); Li *et al.* (2009 ▶); Dutkiewicz *et al.* (2011 ▶). For the various derivatives of 4,4-difluoro­chalcone, see: Fun *et al.* (2010 ▶); Jasinski *et al.* (2010 ▶). For puckering parameters, see: Cremer & Pople (1975 ▶).
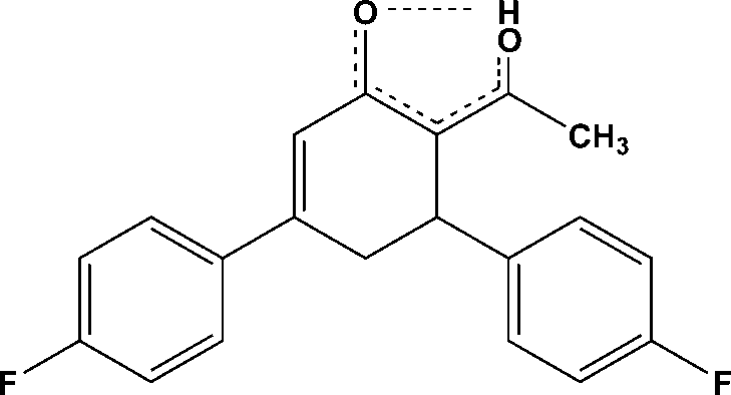


## Experimental

### 

#### Crystal data

C_20_H_16_F_2_O_2_
*M* *_r_* = 326.33
Monoclinic, 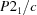

*a* = 17.663 (2) Å
*b* = 6.2371 (6) Å
*c* = 15.2357 (16) Å
β = 107.717 (13)°
*V* = 1598.9 (3) Å^3^
*Z* = 4
Cu *K*α radiation
μ = 0.85 mm^−1^
*T* = 173 K
0.35 × 0.20 × 0.18 mm

#### Data collection

Oxford Diffraction Xcalibur Gemini diffractometer
Absorption correction: multi-scan (*CrysAlis RED*; Oxford Diffraction, 2010 ▶) *T*_min_ = 0.754, *T*_max_ = 0.862
5441 measured reflections
3023 independent reflections
2154 reflections with *I* > 2σ(*I*)
*R*_int_ = 0.020

#### Refinement

*R*[*F* ^2^ > 2σ(*F* ^2^)] = 0.048
*wR*(*F* ^2^) = 0.147
*S* = 1.02
3023 reflections
222 parameters
H atoms treated by a mixture of independent and constrained refinement
Δρ_max_ = 0.17 e Å^−3^
Δρ_min_ = −0.20 e Å^−3^

### 

Data collection: *CrysAlis PRO* (Oxford Diffraction, 2010 ▶); cell refinement: *CrysAlis PRO*; data reduction: *CrysAlis RED*; program(s) used to solve structure: *SHELXS97* (Sheldrick, 2008 ▶); program(s) used to refine structure: *SHELXL97* (Sheldrick, 2008 ▶); molecular graphics: *SHELXTL* (Sheldrick, 2008 ▶); software used to prepare material for publication: *SHELXTL*.

## Supplementary Material

Crystal structure: contains datablock(s) global, I. DOI: 10.1107/S1600536812003078/gg2071sup1.cif

Structure factors: contains datablock(s) I. DOI: 10.1107/S1600536812003078/gg2071Isup2.hkl

Supplementary material file. DOI: 10.1107/S1600536812003078/gg2071Isup3.cml

Additional supplementary materials:  crystallographic information; 3D view; checkCIF report

## Figures and Tables

**Table 1:** Hydrogen-bond geometry (Å, °)

*D*—H⋯*A*	*D*—H	H⋯*A*	*D*⋯*A*	*D*—H⋯*A*
O1⋯H1⋯O2	1.22 (3)	1.28 (3)	2.465 (2)	163 (2)
C8—H8*A*⋯O2^i^	1.00	2.52	3.365 (3)	142
C19—H19*A*⋯O2^ii^	0.95	2.51	3.260 (3)	136

Symmetry codes: (i) 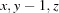; (ii) 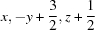.

**Fig. 1.**
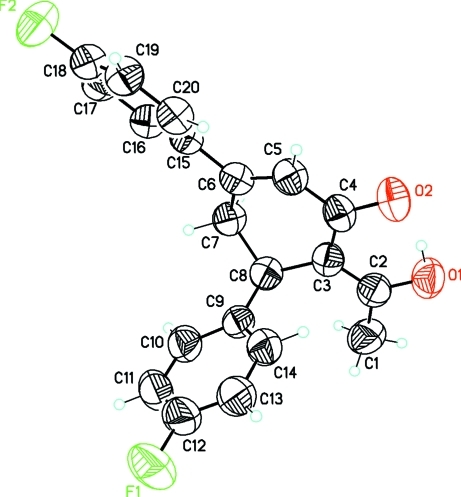
Molecular structure of the title compound showing the atom labeling scheme and 50% probability displacement ellipsoids.

**Fig. 2.**
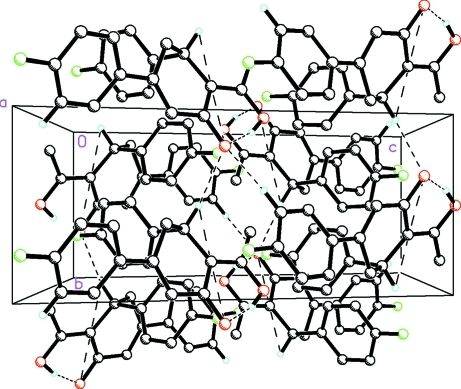
Packing diagram of the title compound viewed along the c axis. Dashed lines indicate O—H···O intramolecular hydrogen bonds and weak C—H···O intermolecular interactions forming an infinite 2-D network along [011]. The remaining H atoms have been removed for clarity.

## supplementary crystallographic information

### Comment

Cyclohexenone derivatives, prepared either from natural sources or entirely via synthetic routes, are known to possess a wide variety of biological activities, e.g. they were reported to have anticonvulsant, antimalarial and cardiovascular effects (Eddington *et al.*, 2000). They are also well known lead molecules for the treatment of inflammation and autoimmune diseases (Kolesnick & Golde, 1994). Cyclohexenones are efficient synthons in building spiro compounds (Padmavathi, Sharmila, Somashekara Reddy & Bhaskar Reddy, 2001) or intermediates in the synthesis of benzisoxazoles or carbazole derivatives (Padmavathi *et al.*, 2000; Padmavathi, Sharmila, Somashekara Reddy & Bhaskar Reddy, 2001; Padmavathi, Sharmila, Balaiah *et al.*, 2001). The crystal structures of some cyclohexenone derivatives viz, rac-ethyl 3-(3-bromo-2-thienyl)-2-oxo-6-(4-propoxyphenyl) cyclohex-3-ene-1-carboxylate (Fischer *et al.*, 2008), ethyl 6-(6-methoxy-2- naphthyl)-2-oxo-4-(2-thienyl)cyclohex-3-ene-1- carboxylate (Li *et al.*, 2009), (1RS,6SR)-Ethyl 4-(2,4-dichlorophenyl)-6-(4-fluorophenyl)-2- oxocyclohex-3-ene-1-carboxylate, (Dutkiewicz *et al.*, 2011) have been reported. In view of the importance of these derivatives and in continuation of our work on the synthesis of various derivatives of 4,4-difluoro chalcone (Fun *et al.*, 2010; Jasinski *et al.*, 2010), the title compound (I) is synthesized and its crystal structure is reported here.

In the title compound, C_20_H_16_F_2_O_2_, the dihedral angle between the mean planes of the cyclohex-2-en-1-one ring (distorted envelope conformation with puckering parameters (Cremer & Pople, 1975) Q, θ and φ of 0.406 (2) Å, 64.7 (3)° and 274.6 (3)°) and the two fluorophenyl rings is 38.9 (8) and 82.3 (1)° (Fig. 1). For an ideal envelope conformation θ and φ are 54.7° and 300°. The two fluorophenyl rings are separated by 77.3 (3)°. The long hydroxyl O–H distance (1.22 (3) Å) in concert with a longer than normal C4=O2 (1.290 (2) Å) bond length suggests a sharing effect between the two oxygen atoms, O1 and O2. Also, with the observation of long C2–C3(1.392 (3)Å) and C4=O2) bond lengths, the influence of an electron delocalization within the O1/C2/C3/C4/O2 moiety may be present. O—H···O intramolecular hydrogen bonds and weak C—H···O intermolecular interactions (Table 1) are observed forming an infinite 2-D network in (011) (Fig. 2).

### Experimental

A mixture of (2E)-1,3-bis(4-fluorophenyl)prop-2-en-1-one (2.44 g, 0.01 mol) and acetyl acetone (1 ml, 0.01 mol) in 20 ml ethanol was refluxed in the presence of a 0.5ml 10% NaOH solution for 6 hours. The reaction mixture was cooled and poured into 50 ml of ice-cold water. The precipitate was collected by filtration and purified by recrystallization from ethanol. Single crystals were grown from dimethylformamide by the slow evaporation method and the yield of the compound was 74%, (m.p. 383 K).

### Refinement

H1 was located by a Fourier map and refined isotropically without restraints. All of the remaining H atoms were placed in their calculated positions and then refined using the riding model with Atom—H lengths of 0.95Å (CH), 0.99Å (CH_2_) or 0.98Å (CH_3_). Isotropic displacement parameters for these atoms were set to 1.2 (CH, CH_2_) or 1.5 (CH_3_) times *U*_eq_ of the parent atom.

### Crystal data

**Table:**

C_20_H_16_F_2_O_2_	*F*(000) = 680
*M**_r_* = 326.33	*D*_x_ = 1.356 Mg m^−^^3^
Monoclinic, *P*2_1_/*c*	Cu *K*α radiation, λ = 1.54178 Å
Hall symbol: -P 2ybc	Cell parameters from 1763 reflections
*a* = 17.663 (2) Å	θ = 3.4–70.8°
*b* = 6.2371 (6) Å	µ = 0.85 mm^−^^1^
*c* = 15.2357 (16) Å	*T* = 173 K
β = 107.717 (13)°	Block, yellow
*V* = 1598.9 (3) Å^3^	0.35 × 0.20 × 0.18 mm
*Z* = 4	

### Data collection

**Table:**

Oxford Diffraction Xcalibur Gemini diffractometer	3023 independent reflections
Radiation source: Enhance (Cu) X-ray Source	2154 reflections with *I* > 2σ(*I*)
Graphite monochromator	*R*_int_ = 0.020
Detector resolution: 16.1500 pixels mm^-1^	θ_max_ = 70.7°, θ_min_ = 5.3°
ω scans	*h* = −21→8
Absorption correction: multi-scan (*CrysAlis RED*; Oxford Diffraction, 2010)	*k* = −7→7
*T*_min_ = 0.754, *T*_max_ = 0.862	*l* = −18→18
5441 measured reflections	

### Refinement

**Table:**

Refinement on *F*^2^	Secondary atom site location: difference Fourier map
Least-squares matrix: full	Hydrogen site location: inferred from neighbouring sites
*R*[*F*^2^ > 2σ(*F*^2^)] = 0.048	H atoms treated by a mixture of independent and constrained refinement
*wR*(*F*^2^) = 0.147	*w* = 1/[σ^2^(*F*_o_^2^) + (0.0646*P*)^2^ + 0.2155*P*] where *P* = (*F*_o_^2^ + 2*F*_c_^2^)/3
*S* = 1.02	(Δ/σ)_max_ < 0.001
3023 reflections	Δρ_max_ = 0.17 e Å^−^^3^
222 parameters	Δρ_min_ = −0.20 e Å^−^^3^
0 restraints	Extinction correction: *SHELXL97* (Sheldrick, 2008), Fc^*^=kFc[1+0.001xFc^2^λ^3^/sin(2θ)]^-1/4^
Primary atom site location: structure-invariant direct methods	Extinction coefficient: 0.0017 (3)

### Special details

**Table:**

Geometry. All esds (except the esd in the dihedral angle between two l.s. planes) are estimated using the full covariance matrix. The cell esds are taken into account individually in the estimation of esds in distances, angles and torsion angles; correlations between esds in cell parameters are only used when they are defined by crystal symmetry. An approximate (isotropic) treatment of cell esds is used for estimating esds involving l.s. planes.
Refinement. Refinement of *F*^2^ against ALL reflections. The weighted *R*-factor *wR* and goodness of fit *S* are based on *F*^2^, conventional *R*-factors *R* are based on *F*, with *F* set to zero for negative *F*^2^. The threshold expression of *F*^2^ > σ(*F*^2^) is used only for calculating *R*-factors(gt) *etc*. and is not relevant to the choice of reflections for refinement. *R*-factors based on *F*^2^ are statistically about twice as large as those based on *F*, and *R*- factors based on ALL data will be even larger.

### Fractional atomic coordinates and isotropic or equivalent isotropic displacement parameters (Å^2^)

**Table:**

	*x*	*y*	*z*	*U*_iso_*/*U*_eq_	
F1	0.55493 (10)	0.1856 (4)	0.43446 (11)	0.1300 (7)	
F2	0.01595 (9)	0.2581 (3)	0.48620 (11)	0.0984 (5)	
O1	0.26793 (10)	0.4978 (3)	−0.05192 (11)	0.0813 (5)	
H1	0.2297 (15)	0.605 (5)	−0.0137 (19)	0.098*	
O2	0.19432 (10)	0.6694 (2)	0.04310 (11)	0.0785 (5)	
C1	0.32971 (15)	0.1596 (5)	−0.01899 (18)	0.0895 (8)	
H1A	0.3318	0.1788	−0.0820	0.134*	
H1B	0.3059	0.0201	−0.0137	0.134*	
H1C	0.3837	0.1657	0.0241	0.134*	
C2	0.28083 (13)	0.3328 (4)	0.00327 (15)	0.0666 (6)	
C3	0.25062 (12)	0.3242 (3)	0.07759 (13)	0.0585 (5)	
C4	0.20637 (13)	0.4997 (3)	0.09387 (14)	0.0610 (5)	
C5	0.17204 (13)	0.4938 (3)	0.16889 (15)	0.0622 (5)	
H5A	0.1539	0.6235	0.1882	0.075*	
C6	0.16517 (11)	0.3107 (3)	0.21179 (14)	0.0552 (5)	
C7	0.19511 (12)	0.1060 (3)	0.18191 (16)	0.0610 (5)	
H7A	0.1511	0.0375	0.1336	0.073*	
H7B	0.2112	0.0069	0.2351	0.073*	
C8	0.26582 (12)	0.1378 (3)	0.14426 (14)	0.0591 (5)	
H8A	0.2699	0.0058	0.1087	0.071*	
C9	0.34407 (12)	0.1584 (3)	0.22286 (14)	0.0598 (5)	
C10	0.37064 (15)	−0.0158 (4)	0.28156 (16)	0.0741 (6)	
H10A	0.3398	−0.1434	0.2722	0.089*	
C11	0.44143 (16)	−0.0060 (5)	0.35355 (18)	0.0892 (8)	
H11A	0.4591	−0.1247	0.3937	0.107*	
C12	0.48489 (16)	0.1786 (6)	0.36503 (17)	0.0879 (8)	
C13	0.46193 (14)	0.3517 (5)	0.31003 (16)	0.0805 (7)	
H13A	0.4936	0.4778	0.3198	0.097*	
C14	0.39064 (13)	0.3403 (4)	0.23873 (15)	0.0692 (6)	
H14A	0.3736	0.4614	0.1999	0.083*	
C15	0.12484 (11)	0.2989 (3)	0.28353 (13)	0.0544 (5)	
C16	0.08461 (12)	0.1136 (4)	0.29494 (15)	0.0623 (5)	
H16A	0.0831	−0.0058	0.2557	0.075*	
C17	0.04699 (12)	0.1000 (4)	0.36192 (16)	0.0674 (6)	
H17A	0.0191	−0.0262	0.3686	0.081*	
C18	0.05082 (13)	0.2727 (4)	0.41839 (15)	0.0680 (6)	
C19	0.08926 (13)	0.4591 (4)	0.41012 (15)	0.0679 (6)	
H19A	0.0906	0.5769	0.4501	0.081*	
C20	0.12583 (12)	0.4709 (3)	0.34258 (14)	0.0616 (5)	
H20A	0.1525	0.5994	0.3359	0.074*	

### Atomic displacement parameters (Å^2^)

**Table:**

	*U*^11^	*U*^22^	*U*^33^	*U*^12^	*U*^13^	*U*^23^
F1	0.0915 (11)	0.195 (2)	0.0799 (10)	0.0137 (13)	−0.0091 (9)	0.0144 (12)
F2	0.1129 (12)	0.1062 (11)	0.0963 (10)	−0.0046 (9)	0.0622 (9)	−0.0015 (9)
O1	0.0858 (11)	0.0952 (12)	0.0606 (9)	−0.0078 (10)	0.0188 (8)	0.0136 (9)
O2	0.0991 (12)	0.0610 (9)	0.0726 (10)	0.0020 (8)	0.0222 (9)	0.0133 (8)
C1	0.0811 (16)	0.114 (2)	0.0818 (16)	0.0050 (16)	0.0377 (14)	0.0018 (16)
C2	0.0587 (12)	0.0782 (15)	0.0583 (12)	−0.0082 (11)	0.0110 (10)	−0.0009 (11)
C3	0.0593 (11)	0.0602 (12)	0.0532 (11)	−0.0066 (9)	0.0130 (9)	−0.0014 (9)
C4	0.0672 (12)	0.0521 (11)	0.0569 (11)	−0.0050 (10)	0.0087 (10)	0.0021 (9)
C5	0.0696 (13)	0.0493 (11)	0.0672 (12)	0.0017 (10)	0.0200 (11)	−0.0023 (10)
C6	0.0528 (10)	0.0474 (10)	0.0614 (11)	−0.0019 (8)	0.0113 (9)	−0.0038 (9)
C7	0.0647 (12)	0.0495 (11)	0.0703 (13)	−0.0029 (9)	0.0225 (10)	−0.0011 (10)
C8	0.0645 (12)	0.0528 (11)	0.0621 (11)	0.0017 (9)	0.0225 (10)	−0.0034 (9)
C9	0.0639 (12)	0.0647 (12)	0.0561 (11)	0.0101 (10)	0.0263 (10)	0.0025 (10)
C10	0.0818 (16)	0.0723 (15)	0.0732 (14)	0.0130 (12)	0.0310 (13)	0.0104 (12)
C11	0.0948 (19)	0.105 (2)	0.0696 (15)	0.0321 (17)	0.0283 (15)	0.0231 (15)
C12	0.0720 (15)	0.130 (2)	0.0589 (13)	0.0129 (17)	0.0151 (12)	0.0062 (16)
C13	0.0727 (14)	0.103 (2)	0.0624 (13)	−0.0063 (14)	0.0154 (12)	0.0002 (14)
C14	0.0668 (13)	0.0773 (15)	0.0614 (12)	−0.0020 (12)	0.0163 (11)	0.0054 (11)
C15	0.0495 (10)	0.0526 (11)	0.0579 (11)	0.0015 (8)	0.0116 (9)	0.0009 (9)
C16	0.0587 (11)	0.0585 (12)	0.0675 (12)	−0.0046 (10)	0.0162 (10)	−0.0061 (10)
C17	0.0566 (12)	0.0648 (13)	0.0809 (14)	−0.0065 (10)	0.0211 (11)	0.0025 (12)
C18	0.0630 (13)	0.0781 (15)	0.0647 (13)	0.0053 (11)	0.0220 (11)	0.0033 (12)
C19	0.0740 (14)	0.0657 (13)	0.0635 (12)	−0.0004 (11)	0.0202 (11)	−0.0071 (11)
C20	0.0633 (12)	0.0546 (11)	0.0626 (12)	−0.0031 (9)	0.0125 (10)	−0.0026 (10)

### Geometric parameters (Å, º)

**Table:**

F1—C12	1.362 (3)	C8—H8A	1.0000
F2—C18	1.357 (2)	C9—C14	1.379 (3)
O1—C2	1.304 (3)	C9—C10	1.394 (3)
O1—H1	1.22 (3)	C10—C11	1.391 (3)
O2—C4	1.290 (2)	C10—H10A	0.9500
O2—H1	1.28 (3)	C11—C12	1.365 (4)
C1—C2	1.485 (3)	C11—H11A	0.9500
C1—H1A	0.9800	C12—C13	1.351 (4)
C1—H1B	0.9800	C13—C14	1.392 (3)
C1—H1C	0.9800	C13—H13A	0.9500
C2—C3	1.392 (3)	C14—H14A	0.9500
C3—C4	1.410 (3)	C15—C16	1.395 (3)
C3—C8	1.513 (3)	C15—C20	1.397 (3)
C4—C5	1.448 (3)	C16—C17	1.380 (3)
C5—C6	1.339 (3)	C16—H16A	0.9500
C5—H5A	0.9500	C17—C18	1.367 (3)
C6—C15	1.477 (3)	C17—H17A	0.9500
C6—C7	1.505 (3)	C18—C19	1.371 (3)
C7—C8	1.538 (3)	C19—C20	1.373 (3)
C7—H7A	0.9900	C19—H19A	0.9500
C7—H7B	0.9900	C20—H20A	0.9500
C8—C9	1.534 (3)		
			
C2—O1—H1	98.0 (12)	C14—C9—C8	123.59 (19)
C4—O2—H1	97.0 (12)	C10—C9—C8	118.9 (2)
C2—C1—H1A	109.5	C11—C10—C9	121.2 (2)
C2—C1—H1B	109.5	C11—C10—H10A	119.4
H1A—C1—H1B	109.5	C9—C10—H10A	119.4
C2—C1—H1C	109.5	C12—C11—C10	118.3 (2)
H1A—C1—H1C	109.5	C12—C11—H11A	120.9
H1B—C1—H1C	109.5	C10—C11—H11A	120.9
O1—C2—C3	121.3 (2)	C13—C12—F1	119.1 (3)
O1—C2—C1	115.2 (2)	C13—C12—C11	122.9 (3)
C3—C2—C1	123.4 (2)	F1—C12—C11	118.0 (3)
C2—C3—C4	118.9 (2)	C12—C13—C14	118.2 (3)
C2—C3—C8	122.8 (2)	C12—C13—H13A	120.9
C4—C3—C8	118.23 (18)	C14—C13—H13A	120.9
O2—C4—C3	122.1 (2)	C9—C14—C13	121.9 (2)
O2—C4—C5	117.5 (2)	C9—C14—H14A	119.1
C3—C4—C5	120.43 (19)	C13—C14—H14A	119.1
C6—C5—C4	121.92 (19)	C16—C15—C20	117.58 (19)
C6—C5—H5A	119.0	C16—C15—C6	120.81 (18)
C4—C5—H5A	119.0	C20—C15—C6	121.61 (18)
C5—C6—C15	122.64 (18)	C17—C16—C15	121.4 (2)
C5—C6—C7	118.91 (19)	C17—C16—H16A	119.3
C15—C6—C7	118.34 (17)	C15—C16—H16A	119.3
C6—C7—C8	113.80 (17)	C18—C17—C16	118.3 (2)
C6—C7—H7A	108.8	C18—C17—H17A	120.9
C8—C7—H7A	108.8	C16—C17—H17A	120.9
C6—C7—H7B	108.8	F2—C18—C17	118.6 (2)
C8—C7—H7B	108.8	F2—C18—C19	118.6 (2)
H7A—C7—H7B	107.7	C17—C18—C19	122.8 (2)
C3—C8—C9	113.18 (17)	C18—C19—C20	118.3 (2)
C3—C8—C7	110.55 (17)	C18—C19—H19A	120.9
C9—C8—C7	111.15 (17)	C20—C19—H19A	120.9
C3—C8—H8A	107.2	C19—C20—C15	121.6 (2)
C9—C8—H8A	107.2	C19—C20—H20A	119.2
C7—C8—H8A	107.2	C15—C20—H20A	119.2
C14—C9—C10	117.5 (2)		
			
O1—C2—C3—C4	−1.1 (3)	C14—C9—C10—C11	0.1 (3)
C1—C2—C3—C4	178.5 (2)	C8—C9—C10—C11	179.6 (2)
O1—C2—C3—C8	−178.63 (18)	C9—C10—C11—C12	−0.5 (4)
C1—C2—C3—C8	1.0 (3)	C10—C11—C12—C13	0.4 (4)
C2—C3—C4—O2	−1.3 (3)	C10—C11—C12—F1	−178.7 (2)
C8—C3—C4—O2	176.34 (18)	F1—C12—C13—C14	179.2 (2)
C2—C3—C4—C5	178.17 (19)	C11—C12—C13—C14	0.1 (4)
C8—C3—C4—C5	−4.2 (3)	C10—C9—C14—C13	0.4 (3)
O2—C4—C5—C6	164.4 (2)	C8—C9—C14—C13	−179.0 (2)
C3—C4—C5—C6	−15.1 (3)	C12—C13—C14—C9	−0.5 (4)
C4—C5—C6—C15	−175.21 (18)	C5—C6—C15—C16	148.4 (2)
C4—C5—C6—C7	0.9 (3)	C7—C6—C15—C16	−27.7 (3)
C5—C6—C7—C8	30.3 (3)	C5—C6—C15—C20	−32.0 (3)
C15—C6—C7—C8	−153.45 (17)	C7—C6—C15—C20	151.90 (19)
C2—C3—C8—C9	85.6 (2)	C20—C15—C16—C17	0.1 (3)
C4—C3—C8—C9	−91.9 (2)	C6—C15—C16—C17	179.70 (18)
C2—C3—C8—C7	−148.96 (19)	C15—C16—C17—C18	−0.9 (3)
C4—C3—C8—C7	33.5 (2)	C16—C17—C18—F2	−178.07 (18)
C6—C7—C8—C3	−45.8 (2)	C16—C17—C18—C19	1.1 (3)
C6—C7—C8—C9	80.8 (2)	F2—C18—C19—C20	178.66 (19)
C3—C8—C9—C14	8.3 (3)	C17—C18—C19—C20	−0.5 (3)
C7—C8—C9—C14	−116.8 (2)	C18—C19—C20—C15	−0.3 (3)
C3—C8—C9—C10	−171.12 (18)	C16—C15—C20—C19	0.5 (3)
C7—C8—C9—C10	63.8 (2)	C6—C15—C20—C19	−179.09 (19)

### Hydrogen-bond geometry (Å, º)

**Table:**

*D*—H···*A*	*D*—H	H···*A*	*D*···*A*	*D*—H···*A*
O1—H1···O2	1.22 (3)	1.28 (3)	2.465 (2)	163 (2)
C8—H8*A*···O2^i^	1.00	2.52	3.365 (3)	142
C19—H19*A*···O2^ii^	0.95	2.51	3.260 (3)	136

Symmetry codes: (i) *x*, *y*−1, *z*; (ii) *x*, −*y*+3/2, *z*+1/2.
